# Vocabulary does not complicate the simple view of reading

**DOI:** 10.1007/s11145-015-9608-6

**Published:** 2015-12-17

**Authors:** David Braze, Leonard Katz, James S. Magnuson, W. Einar Mencl, Whitney Tabor, Julie A. Van Dyke, Tao Gong, Clinton L. Johns, Donald P. Shankweiler

**Affiliations:** Haskins Laboratories, New Haven, CT USA; University of Connecticut, Storrs, CT USA

**Keywords:** Simple view of reading, Adult literacy, Vocabulary, Oral language, Reading comprehension, Structural equation modeling

## Abstract

Gough and Tunmer’s (1986) simple view of reading (SVR) proposed that reading comprehension (RC) is a function of language comprehension (LC) and word recognition/decoding. Braze et al. (2007) presented data suggesting an extension of the SVR in which knowledge of vocabulary (V) affected RC over and above the effects of LC. Tunmer and Chapman (2012) found a similar independent contribution of V to RC when the data were analyzed by hierarchical regression. However, additional analysis by factor analysis and structural equation modeling indicated that the effect of V on RC was, in fact, completely captured by LC itself and there was no need to posit a separate direct effect of V on RC. In the present study, we present new data from young adults with sub-optimal reading skill (*N* = 286). Latent variable and regression analyses support Gough and Tunmer’s original proposal and the conclusions of Tunmer and Chapman that V can be considered a component of LC and not an independent contributor to RC.

## Introduction

The large role of vocabulary in reading ability has long been acknowledged (and extensively assessed) in the reading research literature. Much evidence is consistent with the common-sense assumption that knowledge of word meanings and ready access to that knowledge are causal components of skill in reading comprehension (e.g., Beck & McKeown, 1983; Perfetti, 2007; Perfetti & Stafura, 2014; Senechal, Ouellette, & Rodney, 2006). The question under examination in this study is whether the role of vocabulary should be modeled as distinct from that of general language comprehension ability, when the latter is assessed by measures of listening comprehension.

The simple view of reading (SVR) of Gough and Tunmer (1986) holds that comprehension of language in print, reading comprehension (RC), is the product of just two factors. The first is linguistic comprehension (LC), encapsulating those aspects of knowledge necessary for general understanding of linguistic material and typically measured as ability to comprehend language in the form of sentences or narratives presented to the ear (i.e., listening comprehension). In the SVR, LC is not envisioned as a unitary construct, but as encompassing all those skills and capacities necessary to comprehend *both* the spoken and the printed word, in spite of differences between the linguistic content typically conveyed by print and speech (prosody being a case in point). The second factor is the reader’s ability to recognize or decode printed words (D). D represents the ability to translate the printed word into an internal linguistic code consonant with LC. Gough and Tunmer suggest that the ability to pronounce orthographic pseudowords is the purest measure of D, while conceding that knowledge of the speech-print correspondence rules that support this ability may be insufficient for word recognition in general, especially in the case of a deep orthography like that of English. Thus, LC figures equally in the comprehension of language presented to the ear or to the eye, while D is the new skill that learners must acquire to become readers of their native language, at least to be able to read to the same level as their language comprehension allows. The stronger each component, D and LC, the better the reader will comprehend a text. Conversely, if either component is zero, no reading comprehension is possible.

In the context of the Simple View, vocabulary is just one aspect of LC, and so of equal import to comprehension of print or speech, as with all other aspects of LC. However, some researchers have questioned the assumption that word knowledge contributes identically to comprehension of language in each modality. The question is important, because of the influence that the Simple View continues to have on theoretical proposals regarding the cognitive processes involved in reading and reading skill differences. It is precisely because the SVR has been so influential, having guided or inspired extensive research and theory, that it is essential to test its underlying assumptions.

Braze, Tabor, Shankweiler, and Mencl (2007) presented evidence that suggested a complication for the Simple View. Working with data from a sample of young adults (16–24 years old) living in the northeastern United States of America, they inferred that a reader’s knowledge of vocabulary (V) affects RC directly and independently of LC and D. These findings were based on standard measures of (1) spoken receptive and expressive vocabulary to measure V, (2) word and nonword reading in order to measure D, (3) speech sentence comprehension to measure LC, and (4) printed sentence and passage comprehension to measure RC. Regression models targeting RC with variables representing D, LC and V, showed a significant independent contribution of vocabulary over and above the effects of D and LC. On this evidence, Braze and colleagues proposed that the Simple View be extended to include a separate direct effect of V on RC, in addition to the previously supported direct effects of D and LC. Consistent with the Lexical Quality Hypothesis (Perfetti, 2007), they also suggested that word knowledge, as a top down constraint on comprehension, is more important to comprehension of printed language than to oral language. This, they contend, is due to the referents for speech being often less ambiguous than those for print.

Subsequent work has been inconsistent in its support of the vocabulary-enriched version of the Simple View. Among studies using a linear regression based analytic approach, some have found that measures of vocabulary knowledge capture unique variance in reading comprehension scores, after accounting for the effects of printed word recognition ability and general linguistic knowledge (Fraser & Conti-Ramsden, 2008; Ouellette & Beers, 2010), while others have not (Conners, 2009; Macaruso & Shankweiler, 2010).

More recently, researchers have employed the technique of latent variable modeling to directly assess the question of whether, in the context of the Simple View of Reading, evidence can be found for a latent construct of vocabulary knowledge that is clearly distinct from that of general language skill (Foorman, Herrera, Petscher, Mitchell, & Truckenmiller, 2015; Protopapas, Mouzaki, Sideridis, Kotsolakou, & Simos, 2013; Sabatini, Sawaki, Shore, & Scarborough, 2010; Tunmer & Chapman, 2012). However, all of this work used but a single indicator to measure RC and so may not have thoroughly canvassed the underlying factors identified with reading comprehension (Cutting & Scarborough, 2006; Keenan, Betjemann, & Olson, 2008).

Tunmer and Chapman (2012) presented data for 122 7-year-old children from New Zealand using age-appropriate and culture-appropriate measures of V, D, LC and RC. Regression analysis of their data found a small but significant effect of V on RC over and above the effects of D and LC, as did Braze et al. (2007). However, the authors analyzed their data further using a latent variable approach in order to determine if V clustered with LC, rather than forming its own independent factor (as the regression results suggested). They found that structural models that posited an independent contribution of V to RC fit their data no better than models that lacked an independent factor for V. Thus, they concluded there was no evidence for an independent contribution of V. The fact that hierarchical regression found a small but significant effect of vocabulary measures over that of decoding and oral language comprehension measures was explained as due to a failure of the study’s oral language measures to capture fully all aspects of LC that existed. That is, the specific tests used to measure LC were likely to have been less valid and reliable than were the direct tests of vocabulary knowledge and, because of this, hierarchical regression was able to account for additional covariance between vocabulary and reading comprehension.

Protopapas et al. (2013) reach a similar conclusion to Tunmer and Chapman (2012) based on data from Greek-speaking children of similar age to the English-speaking children that Tunmer and Chapman studied, but who were learning to read a much more transparent orthography. They presented results based on data from a 1 year longitudinal study of 436 Greek school children in grades three to five. Regression analyses targeting concurrent reading comprehension showed that measures of oral language comprehension, decoding skill and vocabulary knowledge all accounted for unique variance in reading comprehension, with vocabulary capturing the largest share at 7.8 %. Measures of oral language and vocabulary also captured unique variance in reading comprehension measured 1 year later, whereas decoding skill did not, regardless of whether concurrent reading comprehension was included as an auto-regressor. They then used structural equation modeling (SEM) to assess whether a conjectured latent variable V mediates the role of LC on RC, or vice versa. They concluded that both models (LC mediating V, and V mediating LC) demonstrate good fits to the data, with neither clearly superior to the other. Thus, their results are consistent with the perspective that the Simple View need not be enriched with an additional component of V.

Still, Protopapas et al. (2013) do point out that the question remains to be answered as to *why* measures of vocabulary so often surface as unique predictors of reading comprehension in regression analyses (e.g., Braze et al., 2007; Fraser & Conti-Ramsden, 2008; Ouellette & Beers, 2010; Tunmer & Chapman, 2012). There are at least two possible answers to this question. It may be that measures of vocabulary and oral language comprehension are indeed reflections of a unitary construct (conventionally labeled LC), but that measures of vocabulary are more robust indicators of that construct than are other measures of oral language skill. Alternatively, it may be that while V and LC are distinct constructs, low reliability of at least some tasks used to measure LC precludes a strong demonstration of divergent validity for those constructs (Protopapas et al., 2013).

The present study was designed to examine the question of the independence of V from LC and D. We largely replicate the analytic methodology of Tunmer and Chapman (2012) and Protopapas et al. (2013), and extend its application to a diverse population of English-speaking adults. We used an age range and sampling procedure designed to capture a sample similar to that studied by Braze et al. (2007). Thus, the present study is a quasi-replication of Tunmer and Chapman, who also worked with readers of English, with the main difference being the age of the reader. Unlike most previous studies (e.g., Protopapas et al., 2013; Tunmer & Chapman, 2012), we also employed multiple indicators of reading comprehension, in order to more thoroughly canvas features of the underlying construct. For adult readers representing a wide range of skill levels, we asked whether there is support for an independent construct, V, or whether the contributions of vocabulary knowledge to reading comprehension are completely subsumed by D and LC (as was found by Tunmer and Chapman for children) when we employ the same analytic methods used by them. This study compared the results of latent variable and multiple regression analyses, as did Tunmer and Chapman.

## Method

### Participants

Participants were 295 native speakers of English, ages from 16 to 25 years (mean 20.18, SD = 2.34). Average years of education was 11.89, SD = 1.71. We recruited through posters placed on adult school and community college campuses and in community gathering places, which by design brought in individuals with a wide range of backgrounds and abilities, including many with low reading scores. Nearly all participants were enrolled in some kind of educational program, whether high school, adult school, or community college. Participants gave informed consent and were paid $100 for completing the procedures described here as well as others. All protocols were approved by the Yale University Human Investigation Committee. Of the 295 participants recruited, nine failed to complete significant portions of the test battery and so were excluded from analysis.

### Measures

Reading comprehension (RC) was measured by the Woodcock-Johnson III reading comprehension subtest (WJ-III, Woodcock, McGrew, & Mather, 2001), possible scores range from 0 to 47, and also by the Gates–MacGinitie level AR reading comprehension subtest, 0–48 possible (MacGinitie, MacGinitie, Maria, Dryer, & Hughes, 2000). A third measure of RC consisted of items from the Reading Comprehension subtest of the Peabody Individual Achievement Test–Revised (PIAT-R; Markwardt, 1998). In order to construct a listening comprehension measure that was comparable to a reading comprehension measure, we split items from the PIAT-R subtest into two parallel sets, leaving one in print for reading and the other presented as speech for listening (Leach, Scarborough, & Rescorla, 2003; Spring & French, 1990). This produced comparable forms of the PIAT, one for reading comprehension (PIAT-Rcomp), 0–41 possible, and one for listening comprehension (PIAT-Lcomp), 0–41 possible. For each abridged form, the standard stop condition of five errors in seven consecutive items was used.

In addition to the PIAT-Lcomp, Listening Comprehension (LC) was measured by the Woodcock-Johnson III oral comprehension subtest, 0–34 possible. Word recognition (D) was measured by the Woodcock-Johnson III subtests for word identification (sight words), 0–76 possible, and word attack (nonword decoding), 0–32 possible, and the TOWRE subtests for word reading fluency and nonword reading fluency (Torgesen, Wagner, & Rashotte, 1999). Possible scores for TOWRE subtests range from 0 to 104 and 0–63, respectively, but because some subjects completed all items with high accuracy in less than the 45 s cut time, scores were converted to rates, items-per-minute. Finally, vocabulary (V) was measured by the Wechsler Abbreviated Scale of Intelligence (WASI; Psychological Corp., 1999) vocabulary subtest, 0–59 possible, and the Peabody Picture Vocabulary Test (PPVT-III; Dunn & Dunn, 1997), 0–204 possible.

Tests were administered individually. Standard administration procedures and instructions were used for all published tests, with the sole exception of the PIAT-R derived sentence comprehension measures (see above). There were two testing sessions of about 3.5 h each, on separate days. Breaks were provided as needed.

## Results

All analyses were carried out using packages contained within the R statistical environment version 2.15.3 (R Core Team, 2013). A summary of raw scores for the indicator variables collected for this study is shown in Table 1. Grade equivalent scores are included to facilitate comparison of our sample with others.^1^ All analyses in this report are based on raw scores. Prior to latent variable analyses and regression modeling, we examined distributions of raw scores for normality and potential outliers. Skewness was observed in most variables and so Box–Cox transformations were applied across the board before further analysis (Box & Cox, 1964). The general form of this family of transformations is:

$$ \hat{y}_{i} = \left\{ {\begin{array}{*{20}l} {\left( {y_{i}^{\lambda } - 1} \right)/\lambda } \hfill & {\left( {\lambda \ne 0} \right)} \hfill \\ {\log \left( {y_{i} } \right)} \hfill & {\left( {\lambda = 0} \right)} \hfill \\ \end{array} } \right. $$

**Table 1:** Summary statistics for untransformed measures

Measures	*N*	Mean	SD	Min	Max	Skew	Lambda
gm.rcomp	224	29.69	9.47	8.00	47.00	−0.0336	0.9213
grade equiv.	224	11.59	2.25	4.10	13.00		
wj3.rcomp	226	33.32	4.27	21.00	43.00	−0.1503	1.3692
grade equiv.	226	8.07	4.39	2.30	19.00		
piat.rcomp	286	26.52	7.69	2.00	41.00	−0.2927	1.2564
grade equiv.	286	6.59	3.15	1.30	14.30		
towre.w	286	118.49	18.50	53.33	190.74	0.3919	0.5838
grade equiv.	286	9.75	2.62	2.20	13.00		
towre.nw	286	57.17	20.65	1.33	110.68	−0.3009	1.1597
grade equiv.	286	7.66	3.60	1.00	13.00		
wj3.watt	286	23.94	6.08	2.00	32.00	−1.0127	2.3171
grade equiv.	286	8.42	4.96	0.30	19.00		
wj3.wid	286	63.05	7.25	36.00	76.00	−0.6285	2.8912
grade equiv.	286	10.16	4.69	2.40	19.00		
piat.lcomp	286	28.59	7.44	9.00	41.00	−0.8300	2.0618
grade equiv.	286	7.42	3.06	1.40	14.30		
wj3.oralcomp	286	23.85	4.34	9.00	33.00	−0.4512	1.8188
grade equiv.	286	10.02	4.71	0.50	19.00		
ppvt	285	159.89	21.24	107.00	198.00	−0.3382	2.1489
wasi.vocab	286	45.89	13.85	12.00	78.00	0.2394	0.6399
age	286	20.18	2.34	16.06	24.98	0.2361	−0.2368
edu	286	11.89	1.71	8.00	17.00	0.6799	−0.5037

Term key: wj3.rcomp, Woodcock Johnson III (WJ3) reading comprehension; piat.rcomp, PIAT-R derived printed sentence comprehension; gm.rcomp, Gates–MacGinitie reading comprehension; towre.nw, TOWRE nonword reading; towre.w, TOWRE word reading; wj3.watt, WJ3 word attack; wj3.wid, WJ3 word identification; piat.lcomp, PIAT-R derived auditory sentence comprehension; wj3.oralcomp, WJ3 oral language comprehension; ppvt, Peabody Picture Vocabulary Test, version 3; wasi.vocab, Weschler’s Abbreviated Scales of Intelligence vocabulary subtest; age, age in years; edu, years of education completed by self-report. See Note 1 for details of grade equivalent scores

Values of lambda were identified that optimize univariate normality for each variable using the *bcpower* function from the *car* package (Fox & Weisberg, 2011). Specific lambdas applied to raw scores are listed in Table 1. Because transformations resulted in large heterogeneity in variances, Box–Cox transformed variables were subsequently standardized.

Inspection of Table 1 shows that there are a number of participants with missing scores on two of our three measures of reading comprehension (Gates–MacGinitie and Woodcock-Johnson reading comprehension subtests). These measures were added to the assessment battery only after data collection had begun. For present purposes, the question arises as to whether the essential characteristics of participants assessed before the additions, and so are missing these scores, are different from those of participants who entered the study afterward. We use the Hawkins test of multivariate normality and heteroscedasticity, as implemented in the R package *MissMech* (Jamshidian & Jalal, 2010; Jamshidian, Jalal, & Jansen, 2014), to address this question. A non-significant *p* value from this test would indicate a lack of sufficient evidence to reject the null hypothesis that data are missing completely at random (MCAR). The method relies on assessing homogeneity of covariances for groups with different patterns of missingness. Our data include five such patterns: 217 complete cases, 54 cases are missing both the WJ3 and Gates reading comprehension tests, six are missing WJ3 reading comprehension only, eight are missing the Gates reading comprehension only, and one is missing the PPVT only. The latter is excluded from the Hawkins test as it requires at least two cases in each group. The Hawkins test indicated there is not sufficient evidence to reject the null hypothesis that missingness in our data set is MCAR (*p* = 0.329).

A correlation matrix of all Box–Cox transformed and standardized variables is presented in Table 2. Inspection of the matrix suggests that there are strong correlations among the variables for V (variables PPVT, WASI vocabulary) and LC (WJ oral comprehension and the PIAT-Lcomp) with smaller correlations between these and the variables for D (WJ word attack, WJ word identification, TOWRE nonwords, and TOWRE words).

**Table 2:** Correlations among variables, after Box–Cox transformation and standardization

	1	2	3	4	5	6	7	8	9	10	11	12
1. gm.rcomp	–											
2. wj3.rcomp	.748	–										
3. piat.rcomp	.662	.689	–									
4. towre.w	.568	.545	.449	–								
5. towre.nw	.519	.543	.461	.765	–							
6. wj3.watt	.561	.610	.535	.605	.812	–						
7. wj3.wid	.703	.727	.657	.657	.761	.798	–					
8. piat.lcomp	.684	.664	.724	.442	.438	.480	.621	–				
9. wj3.oralcomp	.753	.720	.666	.437	.479	.503	.689	.717	–			
10. ppvt	.764	.764	.729	.500	.542	.589	.788	.774	.811	–		
11. wasi.vocab	.729	.701	.662	.483	.533	.557	.738	.698	.757	.805	–	
12. age	.075	-.014	.063	.070	.052	.069	.113	.113	.074	.110	.110	–
13. edu	.248	.275	.271	.268	.314	.279	.367	.331	.316	.403	.387	.464

Term key: wj3.rcomp, Woodcock Johnson III (WJ3) reading comprehension; piat.rcomp, PIAT-R derived printed sentence comprehension; gm.rcomp, Gates–MacGinitie reading comprehension; towre.nw, TOWRE nonword reading; towre.w, TOWRE word reading; wj3.watt, WJ3 word attack; wj3.wid, WJ3 word identification; piat.lcomp, PIAT-R derived auditory sentence comprehension; wj3.oralcomp, WJ3 oral language comprehension; ppvt, Peabody Picture Vocabulary Test, version 3; wasi.vocab, Weschler’s Abbreviated Scales of Intelligence vocabulary subtest; age, age in years; edu, years of education completed by self-report

### Latent variable analysis

All latent variable models were fit with the R package *lavaan* (Rosseel, 2012) using full information maximum likelihood (FIML) as the estimation method. As noted above, manifest variables were Box–Cox transformed and standardized. For the final measurement and structural models we report model χ^2^, Tucker Lewis Index (TLI) and root mean square error of approximation (RMSEA) statistics. The model χ^2^ statistic is an indication of how closely the model-implied covariances reconstruct the empirical covariances. A significant χ^2^ indicates a *poor* match between the two covariance matrices; a non-significant χ^2^ value indicates that there is not a gross mismatch between the two (Kline, 2011). The TLI is a goodness of fit index, penalized for the number of free parameters in the model (Kenny, 2014). The statistic ranges from 0 to 1, with values closer to 1 indicating better fits. The RMSEA, like model χ^2^, is a badness-of-fit statistic; lower values are better. Ideally, the RMSEA will be less than 0.05 and its 90 % confidence interval will include 0 (Kline, 2011).

#### Measurement models

We first tested a series of measurement models that focused on the exogenous variables in the anticipated structural model, with separate factors for V and LC and D. Models that included all four indicators of D in the data set failed to converge, presumably due to high collinearity among the indicators. We eliminated some indicators of D in order to obtain a convergent model. Ultimately, although we define D in terms of nonword reading measures only, defining D with other subsets of our word recognition measures produced similar results (see below). Because the resultant model includes only two measures of D, we constrain their loadings to be equal (Kline, 2011).

**Fig. 1:**
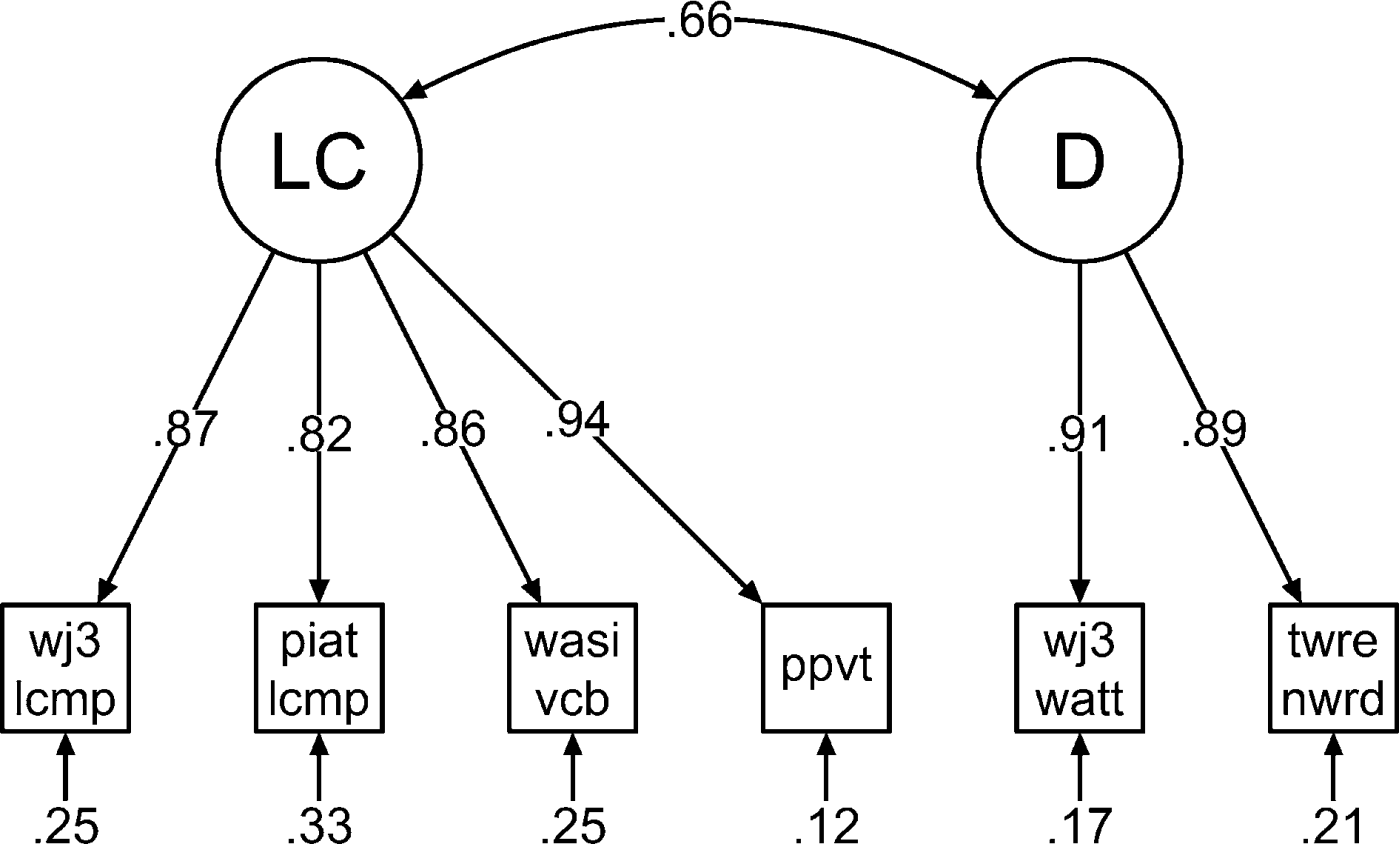
Measurement model including only exogenous latent variables, with standardized coefficients

A subsequent measurement model with separate latent factors for V, LC, and D failed to produce a positive definite matrix, a convergence problem due to the high correlations among the V and LC variables. However, when the variables constituting V and LC were collapsed as a single factor, the measurement model was successful. This model yields a χ^2^(9) = 8.78 (*p* = 0.46), a TLI equal to 1.0 and a RMSEA equal to 0.0 (90 % confidence limits between zero and .065). The model, with standardized coefficients, is depicted in Fig. 1. Interpretation of Fig. 1 is straightforward: the factor coefficients show the vocabulary and listening comprehension manifest variables are well aligned with the LC latent variable, while two decoding manifest variables are consonant with D. Latent variables LC and D are moderately correlated with each other, as would be expected from intercorrelations among their manifest variables seen in Table 2.

The choice of which specific decoding measures to retain in Fig. 1 was made on theoretical grounds, but very similar results were found when we used other subsets of our decoding measures that included WJ word identification and TOWRE words subtests, whose items can be discovered by either sight word recognition or decoding. There were no substantial changes: again, a separate vocabulary factor was not supported. The original model containing only the two nonword reading measures remained numerically strongest. Thus, no matter how D was defined, the main hypothesis of the present study—that vocabulary and listening comprehension are distinct—was clearly refuted.

**Fig. 2:**
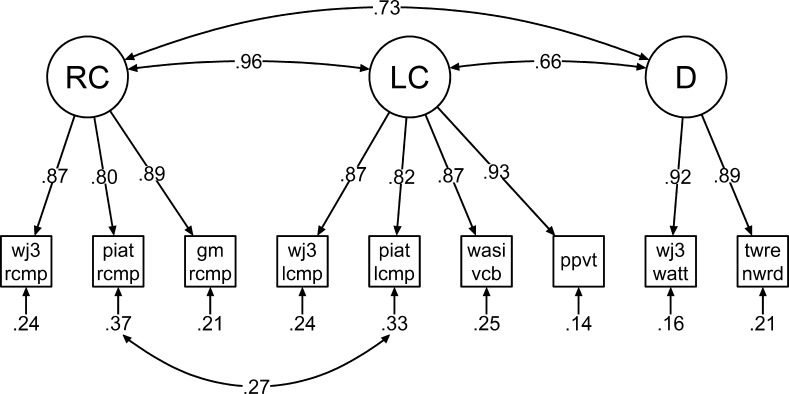
Measurement model including all latent variables, with standardized coefficients

We then took the model in Fig. 1 and added our single endogenous latent variable, RC in order to assess the unity of the manifest variables contributing to the criterion latent factor. In this model we also allowed errors for the manifest variables piat.Lcomp (LC) and piat.Rcomp (RC) to covary. Recall that these measures are derived by splitting items (odd–even) from a single test (Spring & French, 1990; Leach et al., 2003), the PIAT-R sentence comprehension subtest (Markwardt, 1998), and therefore their errors might be expected to covary (Kline, 2011). The resultant model is shown in Fig. 2. The fit was good, with χ^2^(24) = 21.21 (*p* = 0.63), a TLI = 1.0 and RMSEA = 0.0 (90 % CLI between 0.0 and .041). The quality of fit suggests that the model contains a reasonable set of latent constructs, which would support addition of structural components. However, we note that the measurement models in Figs. 1 and 2 already answer our question of whether addition of a V factor to the SVR is supported.

#### Structural equation modeling

**Fig. 3:**
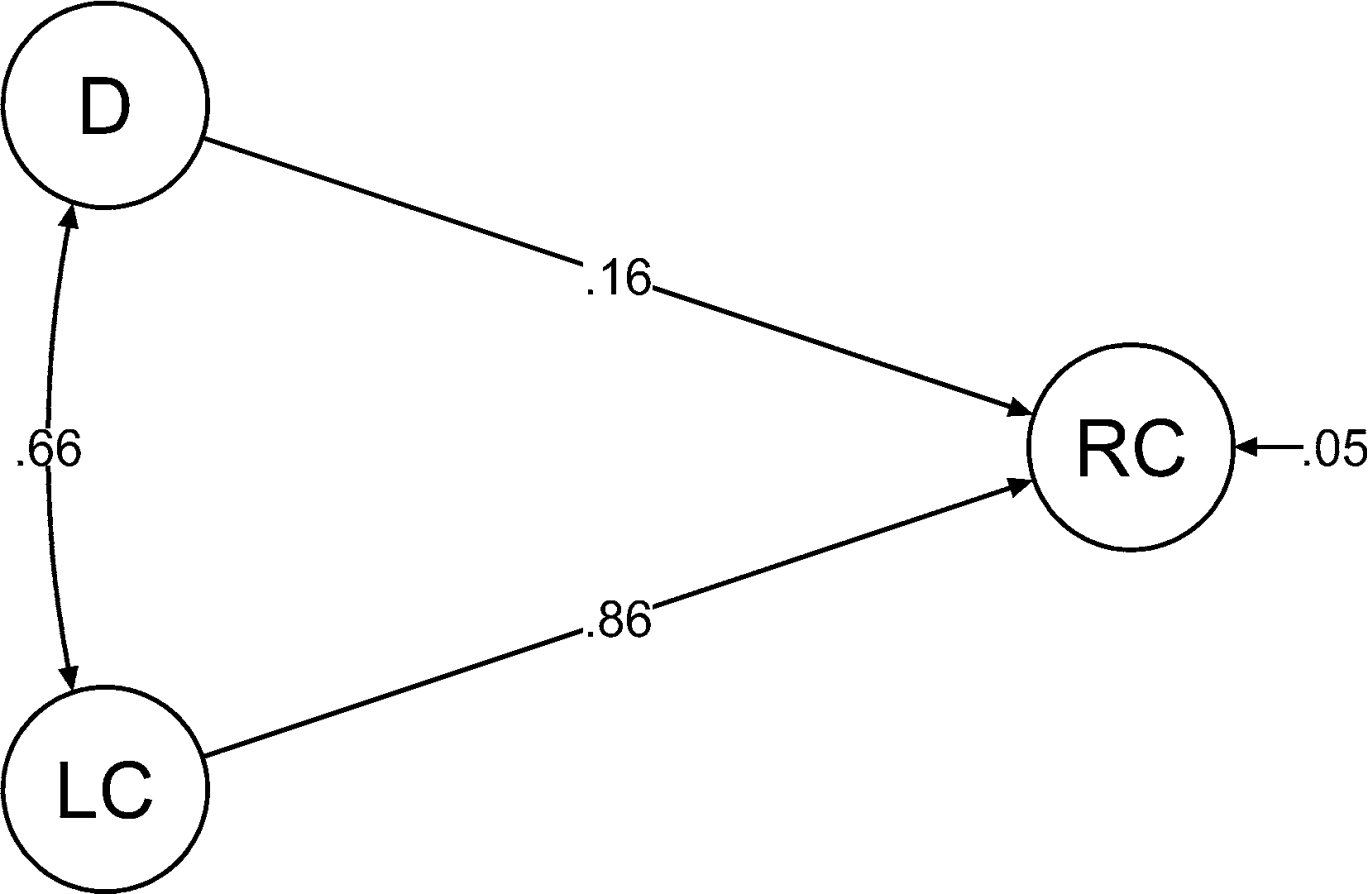
Structural model based on measurement model in Fig. 2, with standardized coefficients

We examined the standard SVR hypothesis that LC and D are determiners of RC. To this end, we conducted an SEM that included the latent variables LC, D, and RC based on the revised measurement model in Fig. 2 (i.e., there is no independent V factor). The structural model, with the resulting path coefficients, is shown in Fig. 3. Table 3, lower triangle, gives the model-implied inter-correlations among all manifest and latent variables for the model in Fig. 3. The model provides a good overall fit to the data, with χ^2^(24) = 21.21 (*p* = 0.63), a TLI = 1.0 and RMSEA = 0.0 (90 % CLI between 0.0 and 0.041). Moreover, its uniformly low residual correlations, shown in the upper triangle of Table 3, suggest that it yields good local fit to the data, across the board.

**Table 3:** Model implied correlations among manifest variables and latent variables that arise from model in Fig. 3 (lower triangle) and correlation residuals (upper triangle, cf. Table 2)

	1	2	3	4	5	6	7	8	9	10	11
1. wj3.rcomp	–	.013	−.001	−.007	−.010	−.008	−.006	.033	−.007		
2. piat.rcomp	.697	–	−.008	−.005	−.001	−.004	.014	−.001	−.057		
3. gm.rcomp	.777	.708	–	.022	.007	.020	−.011	.007	−.013		
4. wj3.oralcomp	.737	.671	.749	–	.000	.001	−.002	−.030	−.036		
5. piat.lcomp	.692	.725	.704	.717	–	−.013	.010	−.020	−.046		
6. wasi.vocab	.730	.665	.742	.756	.711	–	−.001	.029	.023		
7. ppvt	.784	.714	.797	.812	.763	.805	–	.024	−.003		
8. wj3.watt	.588	.536	.598	.533	.500	.528	.567	–	−.002		
9. towre.nw	.568	.518	.577	.515	.483	.510	.547	.814	–		
10. RC	.874	.797	.889	.843	.792	.835	.897	.672	.650	–	
11. LC	.843	.768	.857	.874	.821	.866	.930	.610	.589	.965	–
12. D	.640	.584	.651	.580	.545	.575	.617	.918	.887	.733	.664

Term key: wj3.rcomp, Woodcock Johnson III (WJ3) reading comprehension; piat.rcomp, PIAT-R derived printed sentence comprehension; gm.rcomp, Gates–MacGinitie reading comprehension; towre.nw, TOWRE nonword reading; wj3.watt, WJ3 word attack; piat.lcomp, PIAT-R derived auditory sentence comprehension; wj3.oralcomp, WJ3 oral language comprehension; ppvt, Peabody Picture Vocabulary Test, version 3; wasi.vocab, Weschler’s Abbreviated Scales of Intelligence vocabulary subtest

### Regression analysis

A secondary set of analyses was conducted using linear regression. These were carried out using the *lm* function in the R statistical environment (R Core Team, 2013). Although latent variable modeling has already rejected the notion of V as distinct from LC, our purpose here is to see if the present data are consistent with previous work using regression analysis, which showed that vocabulary measures account for a unique portion of variance in reading comprehension beyond that captured by measures of decoding and oral language skill (Braze et al., 2007; Fraser & Conti-Ramsden, 2008; Ouellette & Beers, 2010; Protopapas et al., 2013; Tunmer & Chapman, 2012). Variables selected for inclusion in the regression analysis are based on the measurement model in Fig. 2. In separate regression models, we targeted each of the three manifest variables used to measure the RC factor in our latent variable models. Manifest variables for LC and D were entered as simultaneous predictors in the three models.

In fact, analysis of the current data set converges with earlier findings that vocabulary measures account for unique variance in reading comprehension even after controlling for the effects of word recognition and listening comprehension (Braze et al., 2007). The proportion of variance uniquely attributable to each vocabulary measure in models targeting the three reading comprehension measures is indicated as their squared semi-partial correlations (SSPC) in Table 4.

**Table 4:** Three regression models targeting separate reading comprehension measures

Term	Estimate	SE	Statistic	*p* value	SSPC
4a: Gates–MacGinitie reading comprehension (*N* = 223; multiple *R* ^2^ = 0.69)					
towre.nw	0.0483	0.0674	0.72	0.4744	0.0007
wj3.watt	0.1443	0.0702	2.06	0.0409	0.0061
piat.lcomp	0.1507	0.0629	2.39	0.0175	0.0083
wj3.oralcomp	0.2956	0.0698	4.23	0.0000	0.0259
ppvt	0.1801	0.0829	2.17	0.0309	0.0068
wasi.vocab	0.2200	0.0776	2.84	0.0050	0.0116
4b: WJ3 reading comprehension (*N* = 225; multiple *R* ^2^ = 0.58)					
towre.nw	0.0076	0.0687	0.11	0.9123	0.0000
wj3.watt	0.2128	0.0712	2.99	0.0031	0.0138
piat.lcomp	0.1167	0.0660	1.77	0.0785	0.0048
wj3.oralcomp	0.2196	0.0712	3.08	0.0023	0.0147
ppvt	0.2796	0.0849	3.29	0.0012	0.0168
wasi.vocab	0.1421	0.0787	1.80	0.0725	0.0050
4c: PIAT-R reading comprehension (*N* = 285; multiple *R* ^2^ = 0.62)					
towre.nw	−0.0689	0.0643	−1.07	0.2846	0.0016
wj3.watt	0.1738	0.0663	2.62	0.0093	0.0094
piat.l	0.3580	0.0605	5.92	0.0000	0.0482
wj3.oralcomp	0.0969	0.0672	1.44	0.1506	0.0029
ppvt	0.2293	0.0802	2.86	0.0046	0.0112
wasi.vocab	0.0916	0.0668	1.37	0.1716	0.0026

Squared semipartial correlation (SSPC) is the increment in variance accounted for when the given term is entered last into the model. Term key: towre.nw, TOWRE nonword reading; wj3.watt, Woodcock Johnson III (WJ3) word attack; piat.lcomp, PIAT-R derived auditory sentence comprehension; wj3.oralcomp, WJ3 oral language comprehension; ppvt, Peabody Picture Vocabulary Test, version 3; wasi.vocab, Weschler’s Abbreviated Scales of Intelligence vocabulary subtest

## Discussion

The result originally reported by Braze et al. (2007), and replicated subsequently by Tunmer and Chapman (2012) and others (see above), was obtained in the present study as well. Regression modeling indicates that measures of vocabulary do capture a small but significant amount of variance in reading comprehension beyond that captured by other oral language measures and decoding measures. However, latent variable analysis does not support the presence of a factor for V, independent of general oral language skill. As Tunmer and Chapman point out, the variance in reading comprehension uniquely captured by vocabulary measures could arise as a result of low reliability in the predictor variables; the non-vocabulary variables used to represent LC may have failed to measure all aspects of LC relevant to RC and so measures of vocabulary may have captured additional aspects of LC not covered by the original variables. Moreover, different measures of RC may draw more or less heavily on various component reading skills, including word knowledge (Cutting & Scarborough, 2006; Keenan et al., 2008).

There is no question that vocabulary is an essential part of general language capacity in both older and younger readers (Joshi, 2005; Perin, 2013). Obviously, understanding will be impaired if a text contains words whose meanings are not known to the reader. However, the issue is more subtle: word knowledge is not all or none, and it is possible to have some knowledge of a word’s meaning and range of use without being able to fully apprehend every nuance. More elaborate lexical representations—that is, higher quality representations—that incorporate subtle gradations of meaning, may integrate more flexibly into representations of discourse or narrative and, as a result may be more readily recognized in context (Braze et al., 2007; Perfetti, 2007).

It seems likely that individual differences in reading comprehension reflect changes in components of reading skill as associated with D (accuracy in word recognition), but also proficiency in dealing with material that contains syntactic or pragmatic challenges, or specialized vocabulary, all of which fall within the scope of LC (Braze et al., 2011; Braze, Shankweiler, Ni, & Palumbo, 2002; Frost et al., 2009; Shankweiler, Mencl, Braze, Tabor, Pugh & Fulbright, 2008; Perfetti & Stafura, 2014). Comparison of relative weights across studies of LC and D in supporting RC hints at a developmental progression. Foorman et al. (2015) reported that in their 1st and 2nd grade cohorts, oral language and decoding skill figure about equally in their contributions to reading comprehension. Tunmer and Chapman (2012) found that D weighed somewhat more heavily than LC in their study of 3rd grade students. It may be worth noting that Foorman and colleagues used a speeded measure of decoding skill, while those used by Tunmer and Chapman were simple accuracy measures. Results from Sabatini et al. (2010) indicate that for adults in an Adult Basic Literacy program, with reading skills in the 3rd to 4th grade range, D and LC were again nearly equal in their contributions to reading comprehension. Finally, our own study examined reading in an adult sample that struggles somewhat with reading, but not nearly to the extent of the Sabatini sample. We found that reading comprehension in this group depends rather more heavily on oral language comprehension than on decoding. Looking across studies, the reading comprehension of better readers seems to be more constrained by limits on their oral language comprehension than on decoding skill, whereas limits on decoding figure more prominently in less skilled readers.

The debate surrounding the status of word knowledge is particularly significant because the vocabulary of school and instruction is not at all the same as the vernacular language. Yet, it is the former that provides entry into the world of higher education, commerce, and industry. The differences are often magnified in predominantly low SES communities (Townsend, Filippini, Collins, & Biancarosa, 2012), and commonly used measures of vocabulary are not designed to capture this distinction. Further, as Braze et al. (2007) maintained, these differences are associated with language modality. Academic language, though it is reflected in both speech and print, is the language of expository print material.

Although we found no evidence of an important separation between vocabulary knowledge and listening comprehension, we do not conclude from these results that oral language comprehension is necessarily the product of a single latent variable, nor even that reading comprehension relies solely on D and LC. Certainly, some evidence points to the decomposability of LC in both beginning readers and struggling older readers (Tighe & Schatschneider, 2015; Foorman et al., 2015). Moreover, the roles of more general cognitive mechanisms must be taken into account, and proposals for relatively language specific extensions to the Simple View of Reading remain part of the discussion in the research community (e.g., Adlof, Catts, & Little, 2006; Foorman et al., 2015; Silverman, Speece, Harring, & Ritchey, 2012). Limitations of instruments used to assess component skills, in terms of measurement reliability and construct validity, may constrain our ability to discriminate among underlying factors and to discern their roles. Some of those limitations are apparent in the observed differences among grade equivalent scores for the measures of RC in the present study (see Table 1).

Thus, although our data indicate that, at least within the context of the Simple View of Reading, there is no basis for treating vocabulary as anything other than a component of listening comprehension, it is clear that the role of vocabulary in reading and its relation to other language skills is not fully understood. Further research is necessary to understand the ramifications of weak vocabulary knowledge and its interaction with other skills necessary to reading comprehension (e.g., Ouellette 2006; Perfetti & Stafura, 2014; Uccelli et al., 2015; Van Dyke et al., 2014). Perhaps most significantly, vocabulary is a skill that we know how to teach; several studies have shown vocabulary to be highly amenable to training (Coyne et al., 2007, 2010; Roberts, Torgesen, Boardman, & Scammacca, 2008; Scammacca, Roberts, Vaughn, & Stuebing, 2015). Moreover, the importance of rich spoken vocabulary experience for preliterate language development is well known (Hart & Risley, 2003). Consequently, training to improve vocabulary knowledge is an important lever that can be used to drive gains in general language comprehension, which has been demonstrated repeatedly to have an extremely high association with reading skill.

## Footnotes

1 Due to the derivative nature of the PIAT-R based sentence comprehension measures, the standard method of computing grade-equivalent scores could not be used. Therefore, per Braze et al. (2007), grade equivalent scores were calculated by first scaling each raw score, *s*, according to the formula: *s2* *+* *18*. This value was then entered into the reading comprehension column of Table G1 in Markwardt (1998). Further, grade equivalent scores for some measures include non-numerical values like “K.3” corresponding to kindergarten year 3rd month, or “>12.6” corresponding to greater than grade 12, 6th month. Where these occur in our data, they are converted to numerical values on the following models: “K.3” becomes 0.3; “>12.6” becomes 13, the next highest integer. Similarly, a PHS (post-high-school) grade equivalent score on the Gates-MacGinitie reading comprehension subtest, the most common score for this subtest in our sample, is converted to 13. These conversions are done solely for the purpose of enabling numerical summaries of grade equivalent scores provided in Table 1. All analyses are based on raw scores.
